# 4-(4-Fluoro­benzo­yl)-3-phenyl-3,4-di­hydro­naphthalen-1(2*H*)-one

**DOI:** 10.1107/S1600536813008829

**Published:** 2013-04-10

**Authors:** Hao Zhang, Yi-Min Hu

**Affiliations:** a School of Chemistry and Materials Science, Anhui Normal University, Wuhu, Anhui 241000, People’s Republic of China

## Abstract

In the title compound, C_23_H_17_FO_2_, the cyclo­hexenone ring has an envelope conformation, the flap atom being the C atom to which the phenyl ring is attached. The 4-fluoro­benzoyl ring and the phenyl ring are inclined to one another by 28.77 (7)°, and by 52.00 (7) and 44.77 (7) °, respectively, to the aromatic ring fused to the cyclo­hexenone ring. In the crystal, mol­ecules are linked *via* C—H⋯O hydrogen bonds, forming a two-dimensional network lying parallel to (100).

## Related literature

For the domino reaction as an important tool in the construction of structurally complicated mol­ecules, see: Zhao *et al.* (2012 ▶). For Pd-catalysed cascade reactions, see: Wang & Hu (2011 ▶); Yu & Hu (2012 ▶). For the use of condensed polycyclic compounds as synthetic building blocks, pharmacophores and electroluminescent materials, see: Rixson *et al.* (2012 ▶). For cross-coupling reactions of aryl halides with olefins and diynes, see: Hu *et al.* (2010 ▶, 2009 ▶).
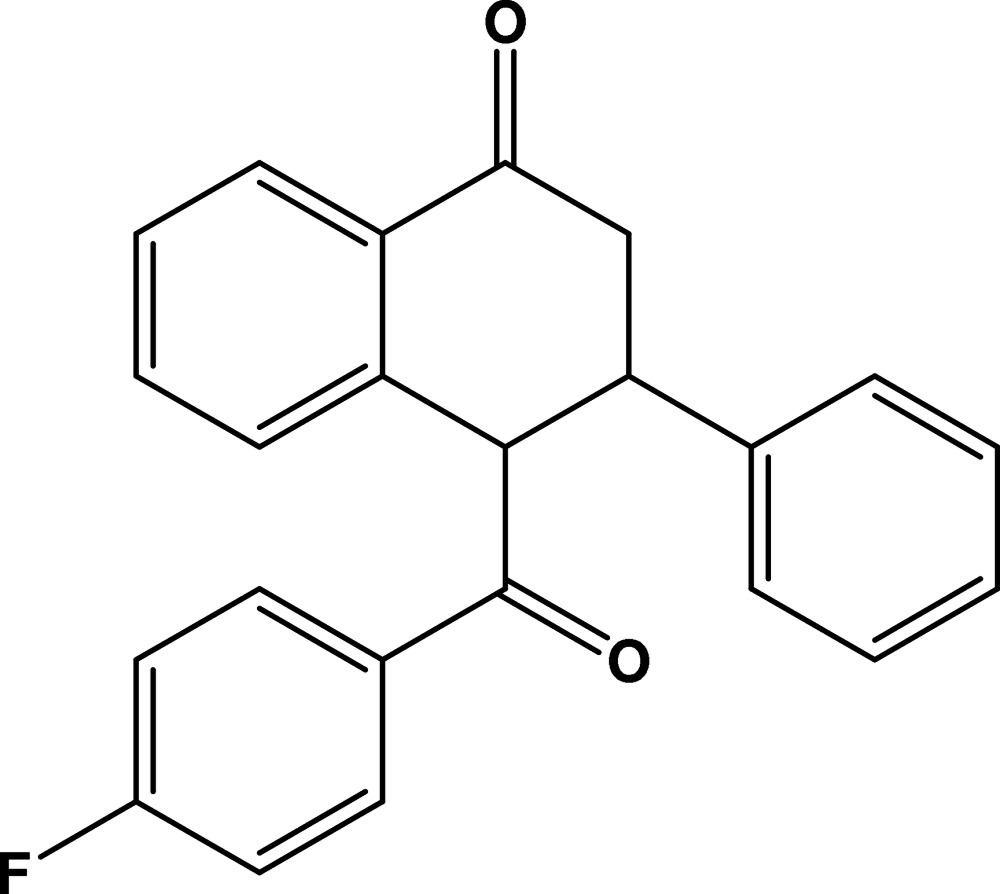


## Experimental

### 

#### Crystal data

C_23_H_17_FO_2_
*M* *_r_* = 344.37
Monoclinic, 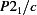

*a* = 8.0063 (6) Å
*b* = 10.6688 (8) Å
*c* = 20.3796 (15) Å
β = 97.458 (1)°
*V* = 1726.1 (2) Å^3^
*Z* = 4
Mo *K*α radiation
μ = 0.09 mm^−1^
*T* = 295 K
0.35 × 0.32 × 0.29 mm

#### Data collection

Bruker SMART APEX CCD diffractometer
Absorption correction: multi-scan (*SADABS*; Bruker, 2000 ▶) *T*_min_ = 0.969, *T*_max_ = 0.974
14632 measured reflections
3987 independent reflections
3176 reflections with *I* > 2σ(*I*)
*R*_int_ = 0.025

#### Refinement

*R*[*F* ^2^ > 2σ(*F* ^2^)] = 0.044
*wR*(*F* ^2^) = 0.117
*S* = 1.03
3987 reflections
235 parameters
H-atom parameters constrained
Δρ_max_ = 0.23 e Å^−3^
Δρ_min_ = −0.28 e Å^−3^

### 

Data collection: *SMART* (Bruker, 2000 ▶); cell refinement: *SAINT* (Bruker, 2000 ▶); data reduction: *SAINT*; program(s) used to solve structure: *SHELXTL* (Sheldrick, 2008 ▶); program(s) used to refine structure: *SHELXTL*; molecular graphics: *SHELXTL*; software used to prepare material for publication: *SHELXTL*.

## Supplementary Material

Crystal structure: contains datablock(s) global, I. DOI: 10.1107/S1600536813008829/rk2398sup1.cif

Structure factors: contains datablock(s) I. DOI: 10.1107/S1600536813008829/rk2398Isup2.hkl

Supplementary material file. DOI: 10.1107/S1600536813008829/rk2398Isup3.cml

Additional supplementary materials:  crystallographic information; 3D view; checkCIF report

## Figures and Tables

**Table 1:** Hydrogen-bond geometry (Å, °)

*D*—H⋯*A*	*D*—H	H⋯*A*	*D*⋯*A*	*D*—H⋯*A*
C14—H14⋯O1^i^	0.93	2.53	3.425 (2)	161
C10—H10⋯O2^ii^	0.98	2.51	3.1427 (15)	123

Symmetry codes: (i) 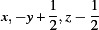; (ii) 
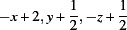
.

**Fig. 1.**
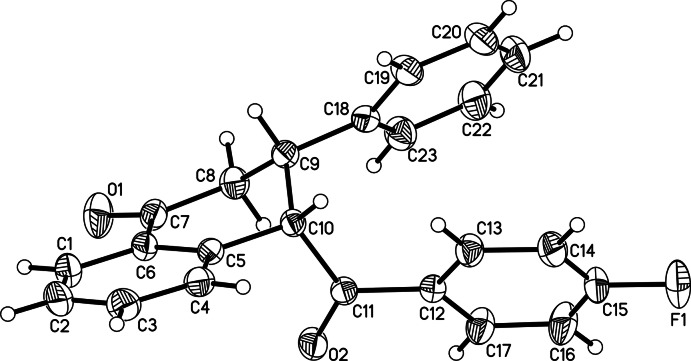
A view of the title compound showing the atom-numbering scheme. Displacement ellipsoids are drawn at 30% probability level. H atoms are presented as a small spheres of arbitrary radius.

**Fig. 2.**
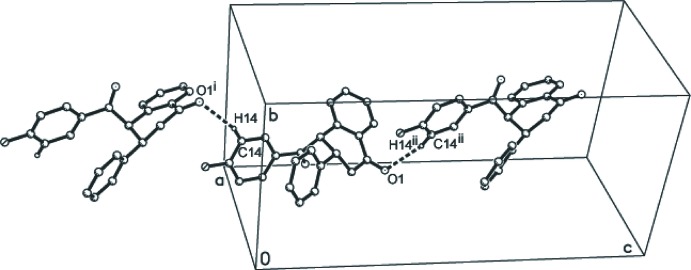
A view of 1-D chain along c axis. Symmetry codes: (i) x, -y+1/2, z-1/2; (ii) x, -y+1/2, z+1/2.

**Fig. 3.**
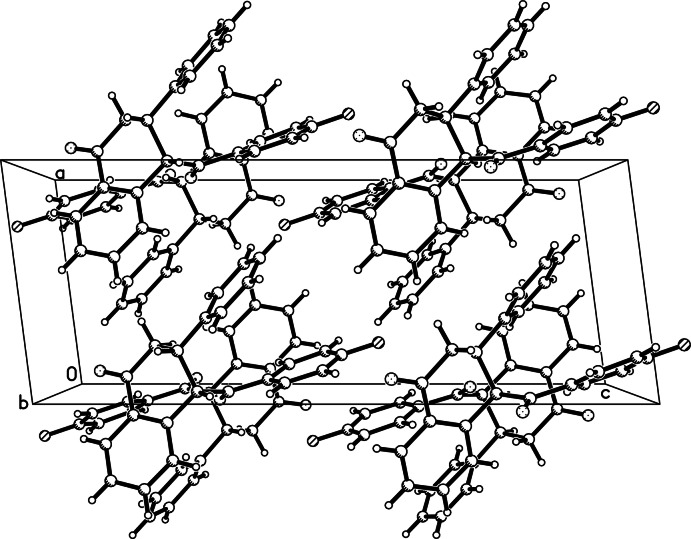
A view of the cell paking down b axis.

## supplementary crystallographic information

### Comment

Domino reaction as an important tool to construct structurally complicate molecule due to high atom economy and environmental benefits (Zhao *et al.*, 2012). Pd-catalyzed cascade reactions have become an efficient protocol of modern organic synthesis chemistry (Wang *et al.*, 2011; Yu *et al.*, 2012). Condensed polycyclic compounds are playing increasingly important roles as synthetic building blocks, pharmacophores, and electroluminescence materials (Rixson *et al.*, 2012). We have reported some novel cross-coupling reactions of aryl halides with the olefins and diynes (Hu *et al.*, 2009; 2010). The reaction of bromobenzene with 1-(2-((4-fluorophenyl)ethynyl)phenyl)prop-2-en-1-one, in the presence of Pd(II) acetate and triphenylphosphine, in *DMF* at 418 K for 19 h, gave the unexpected title product.

The crystal structure data of molecule, C_31_H_30_N_2_O, reveals that all the bond lengths and angles have normal values. The titled molecule contains three phenyl ring and one six-membered carbon ring with a boat conformation. One phenyl ring and the *cis*-fused cyclohexene ring are common side. All the rings are not coplanar (Fig. 1). In the molecule there are two chiral carbon atoms, C9 and C10, but the crystal is a racemic system due to lacking of the chiral separation. In the crystal packing, there are weak intermolecular C–H···O interactions C14–H14···O1^i^ which forms 1-D chain were formed between neighboring molecules along *c* axis (Fig. 2). Symmetry code: (i) *x*, -*y*+1/2, *z*-1/2.

### Experimental

An oven-dried Schlenk flask was evacuated, filled with nitrogen, and then charged with 1-(2-((4-fluorophenyl)ethynyl)phenyl)prop-2-en-1-one (2.51 g, 10 mmol), bromobenzene (1.72 g, 11 mmol), tributylamine (3 ml), P*Ph*_3_ (52.5 mg, 0.2 mmol), Pd(O*Ac*)_2_ (24 mg, 0.1 mol), and *DMF* (10 ml) to give a yellow solution. The reaction mixture was heated at 418 K with stirring. The reaction mixture was cooled to room temperature after 19 h and the resultant yellow-orange mixture was diluted with *Et*_2_O (10 ml). The mixture was washed with H_2_O (15 ml) and the aqueous layer was extracted with *Et*_2_O (20 ml). The combined organic layers were dried (MgSO_4_), filtered, and concentrated *in vacuo*. The crude product was purified by flash column chromatography on silica gel (petroleum ester : *Et*O*Ac* = 9 : 1) and recrystalized from *Et*O*Ac*, yield 2.45 g (71%). Colourless crystals suitable for X-ray diffraction were obtained by recrystallization from a solution of the title compound from ethyl acetate over a period of one week.

### Refinement

All H atoms were positioned geometrically and refined using a riding model with C–H = 0.93Å-0.98Å with *U*_iso_(H) = 1.2*U*_eq_(C).

### Crystal data

**Table:**

C_23_H_17_FO_2_	*F*(000) = 720
*M**_r_* = 344.37	*D*_x_ = 1.325 Mg m^−^^3^
Monoclinic, *P*2_1_/*c*	Mo *K*α radiation, λ = 0.71073 Å
Hall symbol: -P 2ybc	Cell parameters from 2731 reflections
*a* = 8.0063 (6) Å	θ = 2.1–23.6°
*b* = 10.6688 (8) Å	µ = 0.09 mm^−^^1^
*c* = 20.3796 (15) Å	*T* = 295 K
β = 97.458 (1)°	Block, colourless
*V* = 1726.1 (2) Å^3^	0.35 × 0.32 × 0.29 mm
*Z* = 4	

### Data collection

**Table:**

Bruker SMART APEX CCD diffractometer	3987 independent reflections
Radiation source: sealed tube	3176 reflections with *I* > 2σ(*I*)
Graphite monochromator	*R*_int_ = 0.025
φ– and ω–scans	θ_max_ = 27.6°, θ_min_ = 2.0°
Absorption correction: multi-scan (*SADABS*; Bruker, 2000)	*h* = −10→10
*T*_min_ = 0.969, *T*_max_ = 0.974	*k* = −13→13
14632 measured reflections	*l* = −26→24

### Refinement

**Table:**

Refinement on *F*^2^	Primary atom site location: structure-invariant direct methods
Least-squares matrix: full	Secondary atom site location: difference Fourier map
*R*[*F*^2^ > 2σ(*F*^2^)] = 0.044	Hydrogen site location: inferred from neighbouring sites
*wR*(*F*^2^) = 0.117	H-atom parameters constrained
*S* = 1.03	*w* = 1/[σ^2^(*F*_o_^2^) + (0.05*P*)^2^ + 0.44*P*] where *P* = (*F*_o_^2^ + 2*F*_c_^2^)/3
3987 reflections	(Δ/σ)_max_ < 0.001
235 parameters	Δρ_max_ = 0.23 e Å^−^^3^
0 restraints	Δρ_min_ = −0.28 e Å^−^^3^

### Special details

**Table:**

Geometry. All s.u.'s (except the s.u. in the dihedral angle between two l.s. planes) are estimated using the full covariance matrix. The cell s.u.'s are taken into account individually in the estimation of s.u.'s in distances, angles and torsion angles; correlations between s.u.'s in cell parameters are only used when they are defined by crystal symmetry. An approximate (isotropic) treatment of cell s.u.'s is used for estimating s.u.'s involving l.s. planes.
Refinement. Refinement of *F*^2^ against ALL reflections. The weighted *R*-factor *wR* and goodness of fit *S* are based on *F*^2^, conventional *R*-factors *R* are based on *F*, with *F* set to zero for negative *F*^2^. The threshold expression of *F*^2^ > σ(*F*^2^) is used only for calculating *R*-factors(gt) *etc*. and is not relevant to the choice of reflections for refinement. *R*-factors based on *F*^2^ are statistically about twice as large as those based on *F*, and *R*-factors based on ALL data will be even larger.

### Fractional atomic coordinates and isotropic or equivalent isotropic displacement parameters (Å^2^)

**Table:**

	*x*	*y*	*z*	*U*_iso_*/*U*_eq_	
C1	1.2122 (2)	0.13272 (15)	0.42049 (7)	0.0558 (4)	
H1	1.1940	0.0940	0.4598	0.067*	
C2	1.3639 (2)	0.18874 (18)	0.41545 (8)	0.0638 (5)	
H2	1.4479	0.1890	0.4515	0.077*	
C3	1.39238 (19)	0.24486 (16)	0.35687 (8)	0.0590 (4)	
H3	1.4957	0.2826	0.3536	0.071*	
C4	1.26809 (17)	0.24528 (14)	0.30301 (7)	0.0474 (3)	
H4	1.2892	0.2820	0.2635	0.057*	
C5	1.11203 (16)	0.19136 (12)	0.30728 (6)	0.0380 (3)	
C6	1.08446 (17)	0.13349 (12)	0.36657 (6)	0.0429 (3)	
C7	0.92053 (19)	0.07364 (12)	0.37339 (6)	0.0447 (3)	
C8	0.78176 (17)	0.08569 (13)	0.31679 (7)	0.0450 (3)	
H8A	0.6749	0.0903	0.3343	0.054*	
H8B	0.7800	0.0109	0.2896	0.054*	
C9	0.79881 (15)	0.19977 (12)	0.27346 (6)	0.0379 (3)	
H9	0.7963	0.2734	0.3021	0.045*	
C10	0.97521 (15)	0.19869 (11)	0.24892 (6)	0.0346 (3)	
H10	0.9888	0.2775	0.2255	0.041*	
C11	0.98542 (16)	0.09084 (11)	0.20047 (6)	0.0362 (3)	
C12	0.92979 (15)	0.11179 (11)	0.12871 (6)	0.0363 (3)	
C13	0.93535 (19)	0.22788 (13)	0.09884 (7)	0.0466 (3)	
H13	0.9760	0.2966	0.1241	0.056*	
C14	0.8814 (2)	0.24341 (16)	0.03199 (7)	0.0573 (4)	
H14	0.8879	0.3210	0.0117	0.069*	
C15	0.8189 (2)	0.14184 (17)	−0.00295 (7)	0.0574 (4)	
C16	0.8094 (2)	0.02478 (18)	0.02434 (8)	0.0660 (5)	
H16	0.7648	−0.0426	−0.0011	0.079*	
C17	0.8681 (2)	0.00996 (14)	0.09066 (7)	0.0522 (4)	
H17	0.8662	−0.0689	0.1100	0.063*	
C18	0.65496 (16)	0.21523 (12)	0.21773 (6)	0.0400 (3)	
C19	0.62893 (18)	0.33103 (14)	0.18694 (8)	0.0496 (3)	
H19	0.6977	0.3983	0.2016	0.060*	
C20	0.5021 (2)	0.34751 (17)	0.13476 (8)	0.0608 (4)	
H20	0.4877	0.4252	0.1141	0.073*	
C21	0.3973 (2)	0.24981 (19)	0.11325 (8)	0.0655 (5)	
H21	0.3120	0.2612	0.0782	0.079*	
C22	0.4193 (2)	0.13585 (18)	0.14369 (9)	0.0648 (5)	
H22	0.3475	0.0698	0.1297	0.078*	
C23	0.54788 (19)	0.11786 (15)	0.19539 (8)	0.0524 (4)	
H23	0.5624	0.0395	0.2153	0.063*	
F1	0.76491 (16)	0.15640 (12)	−0.06863 (5)	0.0895 (4)	
O1	0.89843 (17)	0.01466 (11)	0.42256 (5)	0.0676 (3)	
O2	1.02932 (15)	−0.01260 (9)	0.22076 (5)	0.0544 (3)	

### Atomic displacement parameters (Å^2^)

**Table:**

	*U*^11^	*U*^22^	*U*^33^	*U*^12^	*U*^13^	*U*^23^
C1	0.0642 (10)	0.0602 (9)	0.0388 (8)	0.0110 (7)	−0.0090 (7)	−0.0028 (6)
C2	0.0542 (9)	0.0783 (11)	0.0523 (9)	0.0133 (8)	−0.0180 (7)	−0.0134 (8)
C3	0.0405 (7)	0.0705 (10)	0.0628 (10)	0.0041 (7)	−0.0052 (7)	−0.0164 (8)
C4	0.0418 (7)	0.0519 (8)	0.0474 (8)	0.0032 (6)	0.0013 (6)	−0.0090 (6)
C5	0.0411 (6)	0.0355 (6)	0.0356 (6)	0.0058 (5)	−0.0025 (5)	−0.0072 (5)
C6	0.0510 (8)	0.0405 (7)	0.0349 (6)	0.0072 (6)	−0.0033 (5)	−0.0044 (5)
C7	0.0633 (9)	0.0361 (7)	0.0339 (7)	0.0038 (6)	0.0028 (6)	−0.0010 (5)
C8	0.0474 (7)	0.0448 (7)	0.0424 (7)	−0.0022 (6)	0.0048 (6)	0.0037 (6)
C9	0.0398 (6)	0.0359 (6)	0.0370 (6)	0.0018 (5)	0.0014 (5)	−0.0020 (5)
C10	0.0396 (6)	0.0301 (6)	0.0327 (6)	0.0003 (5)	−0.0004 (5)	−0.0008 (4)
C11	0.0406 (6)	0.0316 (6)	0.0353 (6)	−0.0001 (5)	0.0007 (5)	−0.0007 (5)
C12	0.0383 (6)	0.0373 (6)	0.0329 (6)	0.0014 (5)	0.0028 (5)	−0.0008 (5)
C13	0.0598 (8)	0.0407 (7)	0.0382 (7)	−0.0004 (6)	0.0020 (6)	0.0027 (5)
C14	0.0731 (10)	0.0558 (9)	0.0422 (8)	0.0100 (8)	0.0050 (7)	0.0127 (7)
C15	0.0601 (9)	0.0781 (11)	0.0311 (7)	0.0101 (8)	−0.0048 (6)	0.0025 (7)
C16	0.0846 (12)	0.0691 (11)	0.0407 (8)	−0.0139 (9)	−0.0061 (8)	−0.0110 (7)
C17	0.0715 (10)	0.0449 (8)	0.0387 (7)	−0.0079 (7)	0.0013 (7)	−0.0022 (6)
C18	0.0362 (6)	0.0441 (7)	0.0395 (7)	0.0054 (5)	0.0036 (5)	−0.0030 (5)
C19	0.0455 (7)	0.0466 (8)	0.0550 (8)	0.0065 (6)	−0.0002 (6)	0.0026 (6)
C20	0.0554 (9)	0.0670 (10)	0.0586 (10)	0.0192 (8)	0.0014 (7)	0.0122 (8)
C21	0.0489 (9)	0.0930 (13)	0.0505 (9)	0.0118 (9)	−0.0088 (7)	−0.0001 (9)
C22	0.0524 (9)	0.0777 (12)	0.0597 (10)	−0.0067 (8)	−0.0104 (7)	−0.0119 (9)
C23	0.0505 (8)	0.0510 (8)	0.0533 (8)	−0.0019 (6)	−0.0032 (6)	−0.0033 (7)
F1	0.1108 (9)	0.1147 (9)	0.0360 (5)	0.0106 (7)	−0.0174 (5)	0.0072 (5)
O1	0.0949 (9)	0.0638 (7)	0.0418 (6)	−0.0137 (6)	0.0003 (6)	0.0132 (5)
O2	0.0832 (8)	0.0338 (5)	0.0426 (5)	0.0118 (5)	−0.0052 (5)	0.0003 (4)

### Geometric parameters (Å, º)

**Table:**

C1—C2	1.369 (2)	C11—C12	1.4892 (17)
C1—C6	1.4009 (19)	C12—C13	1.3834 (18)
C1—H1	0.9300	C12—C17	1.3880 (18)
C2—C3	1.381 (3)	C13—C14	1.3846 (19)
C2—H2	0.9300	C13—H13	0.9300
C3—C4	1.3825 (19)	C14—C15	1.356 (2)
C3—H3	0.9300	C14—H14	0.9300
C4—C5	1.3885 (19)	C15—F1	1.3610 (17)
C4—H4	0.9300	C15—C16	1.373 (2)
C5—C6	1.3997 (19)	C16—C17	1.381 (2)
C5—C10	1.5108 (16)	C16—H16	0.9300
C6—C7	1.482 (2)	C17—H17	0.9300
C7—O1	1.2154 (17)	C18—C23	1.3857 (19)
C7—C8	1.4996 (19)	C18—C19	1.3891 (19)
C8—C9	1.5203 (18)	C19—C20	1.383 (2)
C8—H8A	0.9700	C19—H19	0.9300
C8—H8B	0.9700	C20—C21	1.374 (3)
C9—C18	1.5180 (17)	C20—H20	0.9300
C9—C10	1.5583 (17)	C21—C22	1.366 (3)
C9—H9	0.9800	C21—H21	0.9300
C10—C11	1.5253 (16)	C22—C23	1.388 (2)
C10—H10	0.9800	C22—H22	0.9300
C11—O2	1.2144 (15)	C23—H23	0.9300
			
C2—C1—C6	120.20 (15)	O2—C11—C12	120.48 (11)
C2—C1—H1	119.9	O2—C11—C10	120.17 (11)
C6—C1—H1	119.9	C12—C11—C10	119.22 (10)
C1—C2—C3	120.11 (14)	C13—C12—C17	118.97 (12)
C1—C2—H2	119.9	C13—C12—C11	122.95 (11)
C3—C2—H2	119.9	C17—C12—C11	118.07 (11)
C2—C3—C4	120.35 (15)	C12—C13—C14	121.09 (14)
C2—C3—H3	119.8	C12—C13—H13	119.5
C4—C3—H3	119.8	C14—C13—H13	119.5
C3—C4—C5	120.65 (14)	C15—C14—C13	117.89 (15)
C3—C4—H4	119.7	C15—C14—H14	121.1
C5—C4—H4	119.7	C13—C14—H14	121.1
C4—C5—C6	118.74 (12)	C14—C15—F1	118.27 (15)
C4—C5—C10	119.71 (12)	C14—C15—C16	123.37 (14)
C6—C5—C10	121.53 (12)	F1—C15—C16	118.35 (15)
C5—C6—C1	119.92 (13)	C15—C16—C17	118.08 (15)
C5—C6—C7	120.78 (11)	C15—C16—H16	121.0
C1—C6—C7	119.29 (13)	C17—C16—H16	121.0
O1—C7—C6	121.72 (13)	C16—C17—C12	120.56 (14)
O1—C7—C8	120.39 (14)	C16—C17—H17	119.7
C6—C7—C8	117.88 (11)	C12—C17—H17	119.7
C7—C8—C9	113.70 (11)	C23—C18—C19	117.92 (13)
C7—C8—H8A	108.8	C23—C18—C9	122.78 (12)
C9—C8—H8A	108.8	C19—C18—C9	119.30 (12)
C7—C8—H8B	108.8	C20—C19—C18	120.83 (15)
C9—C8—H8B	108.8	C20—C19—H19	119.6
H8A—C8—H8B	107.7	C18—C19—H19	119.6
C18—C9—C8	113.89 (11)	C21—C20—C19	120.40 (16)
C18—C9—C10	113.07 (10)	C21—C20—H20	119.8
C8—C9—C10	109.51 (10)	C19—C20—H20	119.8
C18—C9—H9	106.6	C22—C21—C20	119.56 (15)
C8—C9—H9	106.6	C22—C21—H21	120.2
C10—C9—H9	106.6	C20—C21—H21	120.2
C5—C10—C11	112.10 (10)	C21—C22—C23	120.46 (16)
C5—C10—C9	110.03 (10)	C21—C22—H22	119.8
C11—C10—C9	109.89 (10)	C23—C22—H22	119.8
C5—C10—H10	108.2	C18—C23—C22	120.82 (15)
C11—C10—H10	108.2	C18—C23—H23	119.6
C9—C10—H10	108.2	C22—C23—H23	119.6
			
C6—C1—C2—C3	−0.8 (2)	C5—C10—C11—C12	−148.71 (11)
C1—C2—C3—C4	0.2 (3)	C9—C10—C11—C12	88.60 (13)
C2—C3—C4—C5	1.1 (2)	O2—C11—C12—C13	−156.49 (14)
C3—C4—C5—C6	−1.8 (2)	C10—C11—C12—C13	27.68 (18)
C3—C4—C5—C10	176.91 (12)	O2—C11—C12—C17	24.2 (2)
C4—C5—C6—C1	1.10 (19)	C10—C11—C12—C17	−151.59 (12)
C10—C5—C6—C1	−177.54 (12)	C17—C12—C13—C14	−0.3 (2)
C4—C5—C6—C7	−179.46 (12)	C11—C12—C13—C14	−179.61 (13)
C10—C5—C6—C7	1.90 (18)	C12—C13—C14—C15	1.7 (2)
C2—C1—C6—C5	0.2 (2)	C13—C14—C15—F1	179.74 (15)
C2—C1—C6—C7	−179.26 (14)	C13—C14—C15—C16	−1.2 (3)
C5—C6—C7—O1	174.25 (13)	C14—C15—C16—C17	−0.5 (3)
C1—C6—C7—O1	−6.3 (2)	F1—C15—C16—C17	178.48 (15)
C5—C6—C7—C8	−4.57 (18)	C15—C16—C17—C12	1.9 (3)
C1—C6—C7—C8	174.87 (13)	C13—C12—C17—C16	−1.5 (2)
O1—C7—C8—C9	156.38 (13)	C11—C12—C17—C16	177.80 (14)
C6—C7—C8—C9	−24.80 (17)	C8—C9—C18—C23	−18.97 (18)
C7—C8—C9—C18	−177.69 (11)	C10—C9—C18—C23	106.89 (15)
C7—C8—C9—C10	54.60 (14)	C8—C9—C18—C19	161.63 (12)
C4—C5—C10—C11	87.24 (14)	C10—C9—C18—C19	−72.51 (15)
C6—C5—C10—C11	−94.14 (14)	C23—C18—C19—C20	−1.3 (2)
C4—C5—C10—C9	−150.15 (12)	C9—C18—C19—C20	178.14 (13)
C6—C5—C10—C9	28.47 (15)	C18—C19—C20—C21	1.2 (2)
C18—C9—C10—C5	176.40 (10)	C19—C20—C21—C22	−0.1 (3)
C8—C9—C10—C5	−55.42 (13)	C20—C21—C22—C23	−0.9 (3)
C18—C9—C10—C11	−59.70 (13)	C19—C18—C23—C22	0.2 (2)
C8—C9—C10—C11	68.48 (13)	C9—C18—C23—C22	−179.17 (14)
C5—C10—C11—O2	35.45 (17)	C21—C22—C23—C18	0.9 (3)
C9—C10—C11—O2	−87.23 (14)		

### Hydrogen-bond geometry (Å, º)

**Table:**

*D*—H···*A*	*D*—H	H···*A*	*D*···*A*	*D*—H···*A*
C14—H14···O1^i^	0.93	2.53	3.425 (2)	161
C10—H10···O2^ii^	0.98	2.51	3.1427 (15)	123

Symmetry codes: (i) *x*, −*y*+1/2, *z*−1/2; (ii) −*x*+2, *y*+1/2, −*z*+1/2.
